# The Dallas School of Pharmacy

**Published:** 1897-09

**Authors:** 


### The Dallas School of Pharmacy.

The JOURNAL has received, as we go to press, the prospectus of the S. W. School of Pharmacy, just organized at Dallas, Texas. The first session will open October 4, 1897. The following are the officers:

DIRECTORS.

Hon. Barnett Gibbs, President; Hon. John H. Traylor, Vice President; Dr. S. H. Stout, Secretary; E. M. Reardon, E. H. R. Green, Royal A. Ferris, H. W. Harry, G. H. Schoellkopf, D. C. Jenkins, T. J. Freeman, W. C. Connor.

FACULTY.

L. Myers Connor, Ph. G., President, Professor of Chemistry.

E. G. Eberle, Ph. G., Vice-President, Professor of Pharmacy and Pharmacognosy.

S. E. Milliken, M. D., Secretary, Professor of Microscopy.

C. M. Rosser, M. D., Professor of Materia Medica.

Q. O. Bradley, Ph. G., Director of Chemical Laboratories.

J. D. Westervelt, Jr., M. D., Director of Microscopical Laboratories.

Ladies are especially invited to study pharmacy. One inducement held out to students, besides the well equipped laboratories, etc., is that there are forty drug stores in Dallas where practical methods may be observed. Dallas was bound to have a college of some sort.

